# Recent Biomarkers for Monitoring the Systemic Fluoride Levels in Exposed Populations: A Systematic Review

**DOI:** 10.3390/ijerph18010317

**Published:** 2021-01-04

**Authors:** Jesús Lavalle-Carrasco, Nelly Molina-Frechero, Martina Nevárez-Rascón, Leonor Sánchez-Pérez, Aida Hamdan-Partida, Rogelio González-González, Diana Cassi, Mario Alberto Isiordia-Espinoza, Ronell Bologna-Molina

**Affiliations:** 1 Dental Sciences, Health Care Department, Autonomous Metropolitan University Xochimilco (UAM), Mexico City 04960, Mexico; lavallec@outlook.com; 2 Health Care Department, Autonomous Metropolitan University Xochimilco (UAM), Mexico City 04960, Mexico; tlsperez@correo.xoc.uam.mx (L.S.-P.); ahamp@correo.xoc.uam.mx (A.H.-P.); 3 Faculty of Dentistry, Autonomous University of Chihuahua (UACH), Chihuahua 31000, Mexico; martina.nevarez@gmail.com; 4 Department of Research, School of Dentistry, Juarez University of the Durango State (UJED), Durango 34000, Mexico; rogegg@hotmail.com (R.G.-G.); ronellbologna@hotmail.com (R.B.-M.); 5 Department of Surgical, Medical, Dental and Morphological Science, University of Modena, 41121 Modena, Italy; dianacassi3@gmail.com; 6 Institute of Research in Medical Sciences, Department of Clinics, Los Altos University Center, University of Guadalajara (UdG), Tepatitlán de Morelos, Jalisco 47650, Mexico; mario.isiordia162@yahoo.com; 7 Molecular Pathology Area, School of Dentistry, University of the Republic (UDELAR), Montevideo 11200, Uruguay

**Keywords:** fluoride biomarkers, recent biomarkers, nails, hair

## Abstract

Fluorides are compounds that can be found in the minerals of soil with volcanic rocks. Different populations are exposed to high levels of fluorides through drinking water that, due to their chronic intake, cause several types of damage to health. Nails and hair, denominated as recent biomarkers, have been employed for monitoring systemic fluoride from long-term exposure to fluorides. The aim of this study was to perform a systematic review of the use of recent biomarkers for monitoring systemic fluoride levels in exposed populations and verify their validity in the measurement of the fluorine (F^−^) concentration within the body. A digital search was performed in the databases PubMed/Medline, Springer Link, Cochrane, and Scopus of original articles that employed recent biomarkers for monitoring systemic F^−^. Seventeen articles were included in this analysis; the recorded variables were the F^−^ amount in each assessed biomarker, source of exposure, and total daily fluoride intake (TDFI). TDFI was associated with F^−^ in nails and hair, as well as the exposure through drinking water. In conclusion, recent biomarkers are adequate for monitoring the systemic fluoride levels by evaluating the chronic/subchronic exposure through different sources, mainly drinking water, considering nails better than hair for this purpose.

## 1. Introduction

Fluorides are compounds formed by the combination of fluorine (F^−^), an electronegative gas, with other elements, and can be found in areas whose soil has high content of volcanic rocks [1,2]. In these areas, populations are at significant risk of health impairment due to the constant exposure to fluorides that leads to a greater consumption of these compounds through several sources [3]. The main source of exposure to fluorides is the drinking water that comes from underground deposits. Fluoride intake provides beneficial effects in the prevention of dental caries when these compounds are found in a concentration between 0.5 and 1.0 mg/L, but when chronically consumed in higher concentrations than recommended, fluorosis, a condition that may occur in animals and humans and affects several organs and systems, can develop, involving subclinical and clinical manifestations [2,4,5].

Around 25 countries in the world are affected by high concentrations of fluorides in drinking water, which comprise 62 million people that are susceptible to developing side effects caused by toxicity [4,5]. Sri Lanka, Ethiopia, Kenya, Iran, China, Mexico, and Argentina, among others, are places where the consumption of grown food from the affected areas adds more intake of fluorides together with the high levels in drinking water [6,7,8,9,10].

Diet is a main source of intake to be considered, since almost all food has a certain amount of fluorides. The levels of these compounds in meat, fruits, and vegetables are usually low. Nonetheless, the concentrations in canned fish like salmon and sardine are elevated, with higher fluoride content in fish from the sea than in those from river water [11,12].

Tea is another important source of fluoride consumption in countries like the Republic of Ireland, New Zealand, and Australia, where it is prepared as an infusion. Meanwhile, in places like India, China, and Egypt, tea is boiled in water, which increases the fluoride concentration [13,14].

Other products that may increase the fluoride intake are dentifrices, drugs, and food with pesticide residues as a result of pollution [15,16].

Due to this public health concern, the World Health Organization has established different biomarkers that have been used to evaluate the exposure to fluorides, such as the historical biomarkers, like bones and teeth, which retain the highest F^−^ quantity in the body; contemporary biomarkers, like blood (plasma), urine, and saliva, which help to detect the levels that are found temporarily after acute intake of fluoride; and finally, those that are called recent biomarkers, which, due to their different advantages, such as obtaining, storage, and processing, have been employed as an emergent and potential alternative for this assessment in contemporary studies [17,18,19,20].

Because there is no consensus regarding which is the most suitable biomarker for assessing the exposure to fluorides, with both nails and hair having been used as novel and emergent biomarkers for this measurement, the aim of the present study was to perform a systematic review of the recent biomarkers that have been employed for monitoring the systemic fluoride levels in exposed populations in association with the sources of intake, and then analyze their biological features, their usefulness in evaluating the exposure to these compounds, and the differences among the rest of the biomarkers. 

## 2. Material and Methods

### 2.1. Protocol and Registration

The protocol of the current systematic review was performed according to the PRISMA guidelines [21], and was previously registered in the database of the International Prospective Register of Systematic Reviews (PROSPERO), with registration number CRD42020167132.

### 2.2. Eligibility Criteria

#### 2.2.1. Inclusion Criteria

The articles that fulfilled the following inclusion criteria were selected: (1) studies that evaluated the exposure to fluorides through recent biomarkers nails and hair, whether by comparing any of these with a different type of biomarker, comparing these biomarkers with each other, or performing the independent evaluation of any of them; (2) articles for which the publication date was from 1 January 2000 to 31 March 2020; (3) studies in the English language; (4) original studies performed on humans.

#### 2.2.2. Exclusion Criteria

The exclusion criteria that were considered for the article selection included the following: (1) studies that evaluated the exposure to fluorides through other biomarkers that did not include those established in the current study; (2) studies that evaluated the exposure to fluorides through recent biomarkers in other animal species; (3) studies that were not originals, such as reviews, letters to editor, or others.

### 2.3. Information Sources

A digital search of the literature was carried out in the main databases, such as PubMed/Medline, Springer link, Cochrane, and Scopus, by using medical subject headings (MeSH) and related terms to recent biomarkers of exposure to fluorides.

### 2.4. Search

The keywords that were employed for the advanced search in each database were: fluoride biomarkers, recent fluoride biomarkers, nails and hair, altogether the Booleans AND, OR, and NOT, using the following terms without abbreviations: “Fluoride biomarkers” OR “recent fluoride biomarkers” AND “nails” OR “hair”, “fluoride biomarkers” OR “recent fluoride biomarkers” AND “nails” NOT “hair”, “fluoride biomarkers” OR “recent biomarkers and fluorides” AND “hair” NOT “nails”. Open access articles were retrieved and those with restricted access were retrieved through institutional access.

### 2.5. Study Selection

First, two independent reviewers performed the screening of title and abstract of each included article according to eligibility criteria. Then, the content of the abstract of each study was analyzed and those articles with relevant information regarding the subject of the current review were selected. Finally, the selected articles were evaluated through full-text analysis in order to determine which of them would be useful for the elaboration of the systematic review. Each reviewer made a list with the names of the articles, which was updated in every described step until defining the final relation of the included studies.

### 2.6. Data Collection Process

The qualitative required data for the study were collected according to standardized forms that contained the most important variables to analyze regarding the subject of the systematic review and the selected articles. This process was performed independently and in duplicate by each reviewer in order to compare the recorded information and correct the differences that were found during this step. In case of disagreement between reviewers, a third reviewer was involved in order to resolve it. Finally, both reviewers completed once again the forms for each study with the corrected information.

### 2.7. Data Items

The variables included and analyzed were: place of the study, year of publication of the study, number of participants, gender, range of age of the participants, study design, employed biomarkers, and sources of exposure to fluorides.

### 2.8. Risk of Bias Assessment

The Cochrane Collaboration’s tool was employed to evaluate risk of bias in the selected studies, which includes the following domains: bias arising from the randomization process, bias due to deviations from intended interventions, bias due to missing outcome data, bias in measurement of the outcome, and bias in selection of the reported results. A description of each domain was made by two independent reviewers according to the information that was evaluated within the studies and a specific grade was given to each domain by using the terms “low risk”, “some concerns”, and “high risk”, as well as an average of the detected risk. Disagreements were discussed, and if needed, were resolved by a third reviewer. The RoB 2 assessment tool was used for the elaboration of the risk of bias figures [22].

### 2.9. Summary Measures

The included measures were the mean and standard deviation (SD) of the F^−^ amount from different sources of exposure to fluorides, range of age of the participants of each study, mean and SD of the measured F^−^ from each analyzed biomarker, and the *p*-value in order to support the significant correlations of the reported results regarding the levels of exposure to fluorides through the considered sources and the biomarkers. 

### 2.10. Synthesis of the Results

The data from each analyzed study were recorded in a database employing the software Microsoft Excel and were organized according to the variables of interest in order to simplify the interpretation and comparison of the results.

## 3. Results

**Figure 1:**
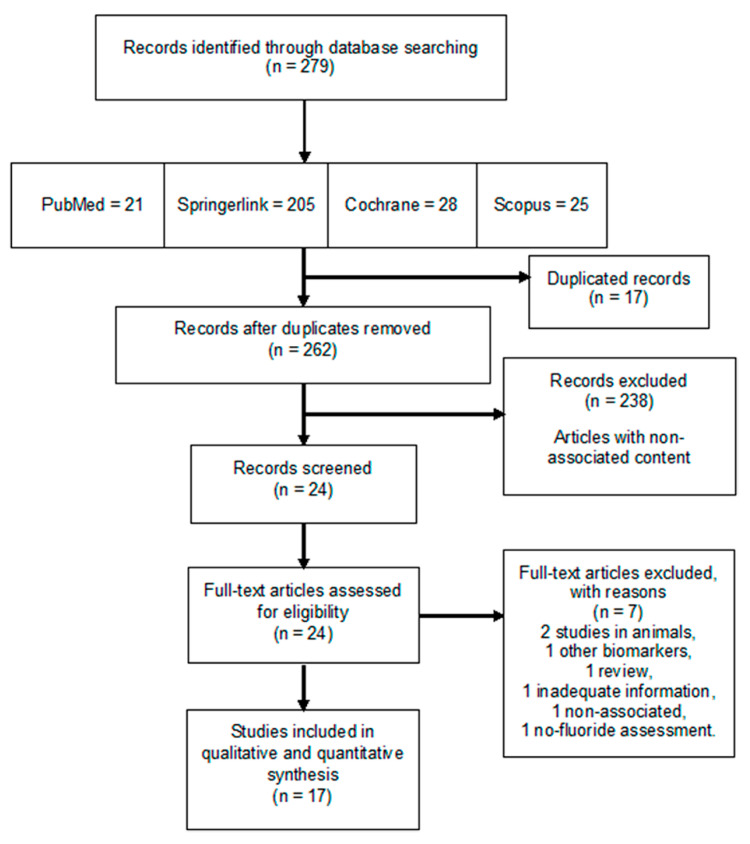
PRISMA flow chart for the systematic review. From the 279 articles found in the four databases included in the search, 17 studies were selected in this study for their analysis.

From the electronic search, a total of 279 articles were registered, of which 17 fulfilled the inclusion criteria after a full-text evaluation, and hence, were included in the systematic review. Figure 1 shows the PRISMA flow diagram with the evaluation and selection process of the chosen studies. 

### 3.1. Risk of Bias 

**Figure 2:**
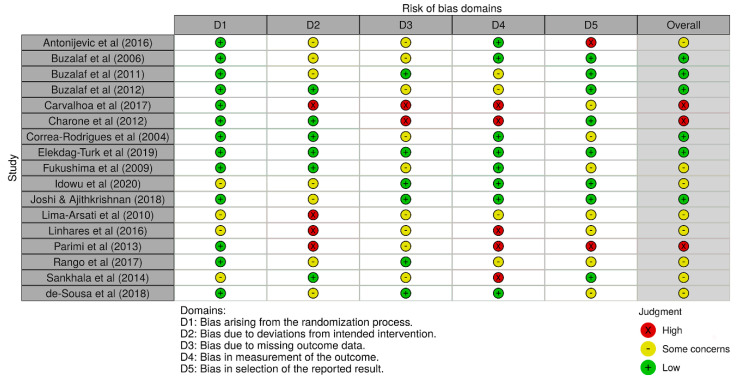
Risk of bias in individual studies. Some concerns of bias predominated in most of the assessed studies, followed by low risk of bias, and finally, high risk of bias.

**Figure 3:**
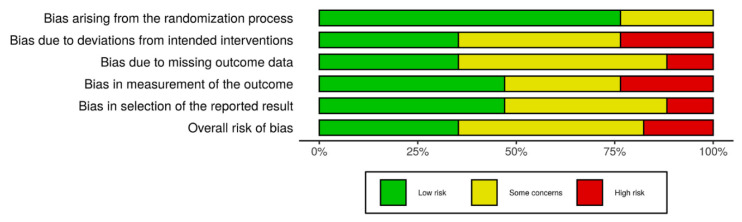
Risk of bias across studies. Excluding the first domain, some concerns and high risk of bias predominated in the rest of them, indicating an important bias by considering the results of all the studies.

Six studies (6/17) presented a low risk of bias, followed by eight studies with some concerns (8/17), and finally, three studies showed high risk (3/17). Figure 2 shows the obtained results of the risk of bias analysis in individual studies according to the evaluated domains. Excluding the first domain (1/6), some concerns and high risk of bias predominated in the assessment of the rest of the domains (5/6), with an overall risk that was over 50% by considering both results. Figure 3 includes the results of the risk of bias across studies analysis, with the given grade for each domain from all the included studies. Despite the result of the risk of bias analysis, the content of the selected articles was relevant for the development of the current review.

### 3.2. Study Characteristics 

From the total of the included articles, 14 (82%) evaluated nails as a biomarker for the F^−^ measurement and 5 (30%) assessed hair. Besides including nails and hair, eight articles (47%) included urine, plasma, or saliva; six studies (35%) employed only nails: of these, two evaluated toenails as well as fingernails, two measured fingernails, and two used toenails; two studies (12%) used hair as a single biomarker; finally, only one study (6%) included exclusively both nails and hair for their comparison.

Most of the studies were published from the year 2010 to 2020 (14/17) and the rest were published from 2000 to 2009 (3/17). The study design of 6 studies was longitudinal (35%) and 11 studies were cross-sectional (65%). Table 1 shows the studies in which the F^−^ levels in fingernails were measured. Table 2 includes the studies in which the F^−^ levels in toenails were quantified. Table 3 presents the studies in which the F^−^ levels in hair were measured. Overall, higher F^−^ levels were observed in the recent biomarkers by considering a higher exposure to fluorides. Moreover, F^−^ content in hair was lower than in nails in those studies that assessed both biomarkers. Table 4 shows the comparison between the main biomarkers employed for monitoring exposure to fluorides and their characteristics. Nails, hair, plasma, saliva, urine, teeth, and bone were included.

## 4. Discussion

Biological human monitoring has been applied as a tool that helps evaluate the health damage in the populations around the world through the evaluation of several biomarkers that provide information on the susceptibility for developing toxic effects as a result of any type of exposure [23]. 

One of the elements that has been widely studied due to its well-known effects in the body is F^−^, whose main source of intake is the naturally fluoridated drinking water for human consumption. When F^−^ is chronically consumed above the recommended concentrations, fluorosis can develop, which is a condition that affects several organs. Teeth are the first tissues damaged, followed by other structures like bones, kidneys, liver, and cardiovascular, reproductive, endocrine, and central nervous systems [2,3,4,24]. 

Diverse biomarkers have been employed in order to assess the exposure to fluorides and the systemic F^−^, and these are divided into different types according to the biological features shared between them [17].

Nails and hair can be highlighted for their capability of reflecting the short-term and long-term exposure to fluorides (from months to years), non-invasive access, and long storage periods without degradation or loss of properties, and because both are supplied by blood during the growth stage, these present the average concentration of plasma fluoride levels from chronic intake [23,25,26,27]. Furthermore, both nails and hair as keratinized matrices share histological features that make them able to attach different types of elements, which is useful for the toxicological evaluation in several research areas [28].

### 4.1. Nails as Biomarkers for Monitoring Fluoride Exposure

Due to the previously described features, nails have been considered as potential biomarkers for monitoring the authentic exposure to fluorides of different populations, mainly through the intake of fluoridated drinking water. According to what was found throughout the systematic review, most of the included studies agree with nails being adequate biomarkers for monitoring chronic/subchronic exposure to these compounds.

The studies performed by Buzalaf et al. demonstrated that nails, specifically toenails, are more sensitive to evaluate subchronic exposure to fluorides. Values tend to increase with age and are associated with the intake of fluoride when these levels are higher than normal, highlighting that it is necessary to consider the growth rate and length together with the exposure time [29,30,31]. 

Corrêa-Rodrigues et al. (2004) agree with the validity of using nails for monitoring subchronic exposure to fluorides from the use of fluoridated dentifrices in children between 2 and 3 years old, adding the consumption of artificially fluoridated water. In this study, no significant differences were found between the F^−^ concentration in fingernails or toenails and the intake of fluorides (*p* = 0.558), but there was a positive significant correlation between both biomarkers (r = 0.571, *p* < 0.01) [32].

The report by Fukushima et al. (2009) highlighted the preferential use of toenails rather than fingernails when monitoring the systemic F^−^ levels in individuals from communities with different fluoride concentrations in drinking water that employed fluoridated dentifrice in distinct age groups. An important finding was that the highest F^−^ concentration was found in the oldest groups from the communities with higher fluoride levels in water, showing significant differences (*p* < 0.001). Moreover, it was pointed out that women tend to have a higher F^−^ concentration in this biomarker [26].

In another study conducted by Linhares et al. (2016), the F^−^ concentration in fingernails of girls and women from different communities was evaluated. Positive significant correlations were found between the measured F^−^ and daily intake, in both girls (r = 0.475, *p* < 0.001) and women (r = 0.495, *p* < 0.001). Thus, the authors concluded that nails are reliable biomarkers for monitoring the systemic F^−^ levels regarding short-term and long-term exposures [33].

Some studies performed the comparison of the exposure to fluorides in more than two biomarkers, including nails, where similar results were found regarding their validity for monitoring systemic F^−^ from different sources of intake.

Idowu et al. (2020) quantified the F^−^ levels in urine, saliva, plasma, hair, fingernails, and toenails that were obtained from children that lived in different communities with low and high concentration of fluorides in drinking water in order to verify the association between exposure and the total daily fluoride intake (TDFI). These authors found a positive significant correlation between TDFI and F^−^ in both fingernails and toenails (*p* < 0.001), with the important difference of toenails presenting a stronger correlation (ρ = 0.604) than fingernails (ρ = 0.470) [34].

A study conducted by Sankhala et al. (2014) compared the F^−^ levels in serum, urine, and toenails in individuals older than 20 years old from communities with high concentration of fluorides in drinking water. Higher F^−^ concentrations were found in toenails in association with high fluoride levels in drinking water, which supports the potential use of this biomarker for monitoring exposure considered as chronic/subchronic [27].

On the other hand, some studies of this review disagree with nails being suitable biomarkers for monitoring the systemic fluoride levels when several sources of intake are considered besides drinking water, or when compared with a biomarker with different features.

Carvalhoa et al. (2017) quantified the F^−^ content of toenail samples obtained from children between 4 and 6 years old in a community with optimal fluoride levels in drinking water. The results showed no significant association between the biomarker and all the considered variables (*p* > 0.05) [35].

De Sousa et al. (2018) used toenails and fingernails for monitoring the systemic F^−^ levels in children from an area where drinking water was naturally fluoridated and another with nonfluoridated drinking water, considering other sources of intake. The main finding of this study pointed out that nails may be employed to reveal this exposure, with significant differences between the F^−^ content in toenails of each community (*p* < 0.05). The authors determined that urine is better for predicting TDFI, unlike nails that did not present a significant association with this variable. Finally, it was concluded that nails are inadequate for monitoring exposure from fluoridated dentifrices [36].

Lima-Arsati et al. (2010) evaluated fingernail samples of children between 1 and 3 years old in order to verify whether these biomarkers were adequate to determine the authentic exposure from the use of fluoridated dentifrice, by considering the fluoride levels in drinking water and diet. No significant differences were found in the biomarker from the period where fluoridated dentifrice was used in comparison to the free fluoridated dentifrice period (*p* = 0.49). Hence, the authors considered that nails may not be a reliable biomarker for monitoring the exposure from the use of fluoridated dentifrices [37].

Rango et al. (2017) evaluated the F^−^ amount in urine and fingernails in individuals between 10 and 50 years old from different communities considering the TDFI from the consumption of drinking water. The authors found significant differences between the fluoride level in the drinking water of each group and the F^−^ content in fingernails (*p* < 0.001), but this was not the same for the age and gender (*p* > 0.05). Furthermore, the authors concluded that urine was a better indicator with a higher sensibility for monitoring immediate exposure [5].

A study conducted by Charone et al. (2012) assessed the fluoride levels in nails in order to find whether the prevalence of dental caries was related or not, without considering any source of exposure. No associations were found between the biomarker and dental caries (*p* > 0.05) [38].

It could be established that nails are adequate as biomarkers for monitoring the systemic fluoride levels from a long-term exposure, although these may not be as sensitive for monitoring exposure from fluoridated dentifrices when its intake is considered solely. Moreover, toenails have demonstrated to be better biomarkers than fingernails, a fact that could be explained by their lower environmental exposure and slower growth rate.

### 4.2. Hair as Biomarker for Monitoring Fluoride Exposure

The findings of the gathered studies in the systematic review revealed promising results regarding the use of hair as a biomarker for evaluating long-term exposure to fluorides when the drinking water is considered as the main source of intake, although this is only reported in the studies where it is employed solely or adding a different type of biomarker.

Antonijevic et al. (2016) obtained hair and urines samples of children from 7 to 15 years old for monitoring the F^−^ levels and determined their relationship to the exposure from different fluoride levels in drinking water. A positive lineal correlation was found in both biomarkers regarding the fluoride levels in drinking water (r = 0.92 in urine and r = 0.94 in hair). Hair was considered a better biomarker for monitoring the exposure to fluorides from drinking water [39].

In a study conducted by Joshi and Ajithkrishnan (2018), hair samples were collected from individuals with a range of age between 35 and 60 years old from endemic and nonendemic fluoride areas in order to assess the F^−^ content. The authors found higher F^−^ levels in hair of individuals from the endemic fluoride area (*p* < 0.001) which proved that this biomarker was adequate for monitoring the chronic exposure to fluorides [40].

This agrees with the report by Parimi et al. (2013) of research performed in individuals between 35 and 60 years old from an area with high and low concentration of fluorides in drinking water, of which the F^-^ content of the obtained hair samples was measured. The biomarker of the participants from the area with high fluoride levels in drinking water had higher F^−^ content in comparison to the control area (*p* = 0.000). Hence, hair was considered adequate for monitoring long-term exposure to fluorides [20].

This information indicates that hair is a useful as biomarker for monitoring and comparing systemic fluoride levels of individuals in areas with high and low exposure to fluorides considering drinking water as the main source of consumption. 

On the other hand, the study performed by Idowu et al. (2020) indicated that hair may not be an adequate biomarker for determining the exposure to fluorides through consumption of drinking water since, in comparison to the rest of the assessed biomarkers, this had the weakest correlation to TDFI (*p* = 0.027, ρ = 0.306) [34]. 

This may indicate that the F^−^ quantified in hair is not as accurate as that present in other biomarkers for monitoring the authentic long-term exposure to fluorides. Thus, it may be necessary to use an additional biomarker to assess this chronic/subchronic exposure, like nails, in order to support the obtained results from hair.

### 4.3. Comparison between Nails and Hair

Only one study, which was performed by Elekdag-Turk et al. (2019), compared exclusively both nails and hair, where the content of F^−^ in the samples of individuals from an endemic and nonendemic fluorosis area was quantified. In both biomarkers, higher F^−^ levels were found in the endemic fluorosis area, showing significant differences (*p* < 0.001 for nails; *p* = 0.004 for hair). Although it was established that nails and hair were adequate biomarkers for monitoring the exposure to fluorides, toenails had more sensitivity for this purpose [25].

This agrees with the report by Idowu et al. (2020), which indicated that toenails were better in comparison to hair for recognizing the most accurate exposure to fluorides [34].

The findings of this review indicate that, despite the similarities of the protein elements that nails and hair have, nails are better biomarkers for monitoring the systemic fluoride levels from a chronic/subchronic exposure, mainly through drinking water consumption and considering TDFI.

### 4.4. Comparison between Recent Biomarkers and the Rest of the Employed Biomarkers of Fluoride Exposure

When comparing nails and hair with the rest of the biomarkers, the first present some advantages that make them an adequate alternative for monitoring the exposure to fluorides (Table 4).

Although historical biomarkers, which include mineralized tissues such as bone and teeth, contain most of the F^−^ burden in the body retained from a long-term exposure, obtaining them is considered invasive because it can only be achieved by surgical procedures. Furthermore, their content can be affected by several events (age, remodeling rate, anatomic site, and genetic background). Contemporary biomarkers, which are fluids such as plasma, saliva, and urine, only reflect the immediate intake of fluorides from a short-term exposure; of these, urine is the most suitable for this purpose, due to the rest of them having some limitations in their use (e.g., saliva is affected by topical F^−^ concentration in the oral cavity and the collection of plasma is considered invasive). Thus, it is necessary to establish the objectives of monitoring the systemic F^−^ levels in individuals from a specific population and to consider what are the conditions to be assessed in order to choose the most suitable biomarker [17,18].

The limitations of this review are the scarce populations that have been assessed by employing the recent biomarkers despite the importance of evaluating the exposure to fluorides, which is reflected in a small number of included studies in this analysis, even in the considered period for the inclusion of the found articles from the electronic search. This was more evident in the use of hair as the main biomarker in comparison to nails, which could be due to the acceptance of researchers and the study individuals regarding the advantages of using nails. Thus, it is necessary to develop further studies in this area that can support the information that is reported so far, and then, provide more validity to the use of the recent biomarkers.

## 5. Conclusions

Nails and hair, as recent biomarkers, are the most adequate elements for monitoring the systemic fluoride levels in association with chronic/subchronic exposure through different sources, mainly the drinking water for human consumption, and considering the TDFI. The findings of this study indicate that nails, specifically toenails, are better than hair and the rest of the known biomarkers for this evaluation due to their biological features, ease of collection, processing, and reliable F^−^ levels recorded from their measurement.

The relevance of these findings focuses on the selection of the most adequate biomarker for the assessment of the exposure to fluorides. Thus, nails and hair should be considered as the first choice in further studies that aim to monitor the authentic long-term exposure to fluorides and the developed damage to health.

## Figures and Tables

**Table 1:** Fluoride concentration in fingernails.

Author	n	M	F	Age (yrs)	Design	Fluoride Exposure ± SD	Mean F^−^ ± SD (µg/g)	*p*-Value
Buzalaf et al. (2006)	10	NS	NS	20–35	Longitudinal	a 0.6	1.48	<0.01
						b, c 1.626 ± 0.774		
Buzalaf et al. (2011)	121	NS	NS	4–6	Cross-sectional	d 0.6–0.8	2.11 ± 0.90	<0.001
						b, c 0.065 ± 0.03		
						d 0.6–0.9	3.38 ± 1.98	
						b, c 0.084 ± 0.029		
						b, c, e 0.088 ± 0.044	6.09 ± 2.21	
						f 250/1.0	2.62 ± 0.82	
						b, c 0.088 ± 0.052		
						b, c 0.027 ± 0.022	1.93 ± 0.82	
Buzalaf et al. (2012)	56	30	26	2–7 10–15	Longitudinal	d 0.1	1.75 ± 0.46	<0.001
						d 0.6–0.8	3.01 ± 1.36	
						d 2.3	6.28 ± 2.69	
Corrêa-Rodrigues et al. (2004)	10	NS	NS	2–3	Longitudinal	d 0.6–0.8 c 1571	2.71 ± 0.97 (for the first 4 weeks and the last 6 weeks)	<0.01
Fukushima et al. (2009)	300	NS	NS	3–7 14–20 30–40 50–60	Longitudinal	d 0.09 ± 0.01, c *	1.54 ± 0.10	<0.001
						d 0.15 ± 0.01, c *	1.54 ± 0.10	
						d 0.66 ± 0.01, c *	1.93 ± 0.15	
						d 0.72 ± 0.02, c *	6.04 ± 0.84	
						d 1.68 ± 0.08, c *	6.08 ± 0.54	
						Mean	3.55 ± 0.24	
Idowu et al. (2020)	61	NS	NS	4–6	Cross-sectional	d 0.04	3.237 ± 2.636	<0.001
						d 3.05	10.420 ± 3.761	
Lima-Arsati et al. (2010)	23	10	3	1–3	Longitudinal	d 0.72 b, c 0.086 ± 0.032		
							2.57 ± 1.49	0.165
						b 0.040 ± 0.009	3.33 ± 1.41	0.701
Linhares et al. (2016)	129	0	129	4–12 25–50	Cross-sectional	d 0.84 ± 0.012	4–12 yrs 2.47 ± 0.27 25–50 yrs 1.40 ± 0.08	<0.05
						d 0.29 ± 0.001	4–12 yrs 1.27 ± 0.33 25–50 yrs 0.67 ± 0.11	
						d 0.43 ± 0.014	4–12 yrs 1.14 ± 0.19 25–50 yrs 1.43 ± 0.44	
						d 1.71 ± 0.008	4–12 yrs 2.78 ± 0.55 25–50 yrs 2.75 ± 0.57	
Rango et al. (2017)	386	NS	NS	10–50	Cross-sectional	d	5.1 ± 4.3	<0.001
						d < 2	2.6 ± 1.6	
						d > 2–5	3.8 ± 2.3	
						d > 5–8	6.8 ± 5.8	
						d > 8–12	7.3 ± 4.4	
de Sousa et al. (2018)	30	NS	NS	2–6	Longitudinal	d, g 0.181 ± 0.132	2.87 ± 1.08	0.24
						d 0.03 ± 0.04 g 0.057 ± 0.045	1.82 ± 0.85	

M: Male; F: Female; yrs: Years old; F^−^; Fluorine; SD: Standard deviation; NS: Not specified; a: Fluoridated solution NaF (mg/mL); b: Diet (mg/kg); c: Dentifrice (mg/kg); d: Naturally fluoridated water (mg/L); e: Fluoridated salt; f: Fluoridated milk (ml/mg/L); g: Total daily fluoride intake (mg/kg/day). * 1500 mg/kg.

**Table 2:** Fluoride concentration in toenails.

Author	n	M	F	Age (yrs)	Design	Fluoride Exposure ± SD	Mean F^–^ ± SD (µg/g)	*p*-Value
Buzalaf et al. (2006)	10	NS	NS	20–35	Longitudinal	a 0.6 b, c 1.626 ± 0.774	1.53	<0.05
Buzalaf et al. (2011)	121	NS	NS	4–6	Cross-sectional	d 0.6–0.8 b, c 0.065 ± 0.03	1.42 ± 0.54	<0.001
						d 0.6–0.9 b, c 0.084 ± 0.029	2.24 ± 0.82	
						b, c, e 0.088 ± 0.044	6.70 ± 3.03	
						f 250/1.0 b, c 0.088 ± 0.052	2.63 ± 0.58	
						b, c 0.027 ± 0.022	1.62 ± 0.47	
Carvalhoa et al. (2017)	242	107	135	4–6	Cross-sectional	d 0.6–0.8	1.61 ± 1.06	>0.05
						g	DF = 1.65 ± 1.08 no DF = 1.58 ± 1.06	
						h	DF = 1.90 ± 1.30 no DF = 1.59 ± 1.04	
						c 1571	DF = 1.87 ± 1.37 no DF = 1.55 ± 0.96	
						i	DF = 1.55 ± 1.17 no DF = 1.63 ± 0.96	
Charone et al. (2012)	54	NS	NS	3–14	Cross-sectional	d	1.85 ± 1.32 1.58 ± 0.78	0.38
Corrêa-Rodrigues et al. (2004)	10	NS	NS	2–3	Longitudinal	d 0.6–0.8 c 1571	2.70 ± 1.17 (for the first 4 weeks and the last 6 weeks)	<0.01
Elekdag-Turk et al. (2019)	42	22	20	NS	Cross-sectional	d ≥ 2.0 d ≤ 0.05	2.34 ± 0.26 0.98 ± 0.08	<0.001
Fukushima et al. (2009)	300	NS	NS	3–7 14–20 30–40 50–60	Longitudinal	d 0.09 ± 0.01, c *	1.14 ± 0.07	<0.001
						d 0.15 ± 0.01, c *	2.27 ± 0.13	
						d 0.66 ± 0.01, c *	1.98 ± 0.12	
						d 0.72 ± 0.02, c *	4.46 ± 0.32	
						d 1.68 ± 0.08, c *	5.94 ± 0.35	
						Mean	3.16 ± 0.14	
Idowu et al. (2020)	61	NS	NS	4–5	Cross-sectional	d 0.04	3.378 ± 2.19	<0.001
						d 3.05	10.371 ± 3.90	
Sankhala et al. (2014)	40	23	17	41–60	Cross-sectional	d 4.1	82.38 ± 6.89	0.001
						d 4.8	86.14 ± 9.77	
						d 5.6	103.92 ± 16.89	
de Sousa et al. (2018)	30	NS	NS	2–6	Longitudinal	d, j 0.181 ± 0.132	2.85 ± 1.35	<0.05
						d 0.03 ± 0.04	1.46 ± 0.27	
						j 0.057 ± 0.045		

M: Male. F: Female. F-: Fluorine. yrs: Years old. SD: Standard deviation. NS: Not specified; DF: Dental fluorosis. a: Fluoridated solution NaF (mg/mL); b: Diet (mg/kg); c: Dentifrice (mg/kg); d: Naturally fluoridated water (mg/L); e: Fluoridated salt; f: Fluoridated milk (ml/mg/L); g: Soy; h: No milk consumption; i: Supervised tooth brushing; j: Total daily fluoride intake (mg/kg/day). * 1500 mg/kg.

**Table 3:** Fluoride concentration in hair.

Author	n	M	F	Age (yrs)	Design	Fluoride Exposure ± SD	Mean F^−^ ± SD (µg/g)	*p*-Value
Antonijevic et al. (2016)	52	29	23	7–15	Cross-sectional	a 0.59	5.28	<0.05
Elekdag-Turk et al. (2019)	42	22	20	NS	Cross-sectional	a ≥ 2.0	0.24 ± 0.04	0.004
						a ≤ 0.05	0.14 ± 0.04	
Idowu et al. (2020)	61	NS	NS	4–5	Cross-sectional	a 0.04	0.74 ± 0.61	0.027
						a 3.05	1.83 ± 1.09	
Joshi & Ajithkrishnan (2013)	36	19	17	35–60	Cross-sectional	a 0.11	0.35 ± 0.063	<0.001
						a 3.43	3.40 ± 1.043	
Parimi et al. (2013)	30	19	11	35–60	Cross-sectional	a 5.2	2171 ± 1647	0.000
						a 0.3–0.5	25.06 ± 18.52	

M: Male; F: Female; F^−^: Fluorine. yrs: Years old; SD: Standard deviation. a: Naturally fluoridated water (mg/L).

**Table 4:** Characteristics of the biomarkers employed for monitoring exposure to fluorides.

Biomarker	Type			Obtaining Method		Storage and Processing		Type of Measured Exposure	
	Recent	Contemporary	Historical	Noninvasive	Invasive	Simple	Complex	Immediate/ Acute	Chronic/ Sub-chronic
Nails	X			X		X			X
Hair	X			X		X			X
Plasma		X			X		X	X	
Saliva		X		X			X	X	
Urine		X		X		X		X	
Teeth			X		X		X		X
Bone			X		X		X		X

## Data Availability

Not applicable.
